# Supplementary material for: Chitin-Derived AVR-48 Prevents Experimental Bronchopulmonary Dysplasia (BPD) and BPD-Associated Pulmonary Hypertension in Newborn Mice
Source: Int J Mol Sci. 2021 Aug 9;22(16):8547. doi: 10.3390/ijms22168547 (PMC8395179; doi:10.3390/ijms22168547)
Supplement: Supplementary file 1 [file ijms-22-08547-s001.zip › ijms-1251655-supplementary.pdf]

Chitin-derived AVR-48 prevents Experimental Bronchopulmonary Dysplasia (BPD) and BPD-associated Pulmonary Hypertension in Newborn Mice

Pragnya Das[1,2,#‡], Suchismita Acharya[3,4#], Varsha M Prahaladan[1,2,‡], Ogan K Kumova[5], Shadi Malaeb[2], Sumita Behera[3], Beamon Agarwal[6], Dale J. Christensen[7,8], Alison J Carey[2,5] and Vineet Bhandari[1,2,*‡]

[1]Department of Pediatrics, Division of Neonatology, Cooper University Hospital, Camden, NJ

[2]Department of Pediatrics, Division of Neonatology, Drexel University, Philadelphia, PA

[3]AyuVis Research, Inc., 1120 South Freeway, Fort Worth, TX

[4]Pharmacology & Neuroscience, University of North Texas Health Science Center, Fort Worth, TX

[5]Department of Microbiology & Immunology, Drexel University, Philadelphia, PA

[6]GenomeRxUS, Secane, PA

[7]Dale J. Christensen Consulting LLC, Cary, NC

[8]Duke University Medical Center, Department of Medicine, Division of Hematology, Durham, NC

Running title: N*ovel chitin derived small molecule for prevention of BPD and BPD-PH*

*****Corresponding author: bhandari-vineet@cooperhealth.edu

The Children's Regional Hospital at Cooper, Dorrance Suite# 755, 1 Cooper Plaza, Camden, NJ 08103,

Tel: 856-342-2000 extn: 1006156; Fax: 856-342-8007

[‡]Current affiliation: Cooper University Health Care and Hospital, Suite# 206, 401 Haddon Avenue, Education and Research Building, Camden, NJ 08103

[#]Equal contributors

Supplemental Methods

Histology of visceral organs

Room air (P7) pups were injected with 100mg/kg AVR-48, IP once a day for 7 consecutive days, following which the lungs, kidney, heart, liver, gut and spleen were harvested and processed for paraffin sectioning and hematoxylin-eosin (H&E) staining following standard procedures.

Fabrication of AVR-48 nanoparticles (AVR-48 NP)

The fabrication procedure was similar to our previous report [1]. In brief, 10mg of AVR-48 was dissolved in 10µl DMSO and transferred to 3ml of chloroform containing 90mg of PLGA to form an oil phase. This solution was then added drop wise to 20ml of 5% PVA solution (water phase) followed by sonication at 40W for 10 minutes to form the AVR-48 loaded NP. The emulsion was stirred overnight to completely evaporate the organic solvent. Next, the NPs were pelleted by ultracentrifugation at 25,000 rpm for 30 minutes followed by washing twice with deionized (DI) water. Finally, the NP pellet was dissolved in DI water and lyophilized to obtain a powder form of AVR-48 NP.

Characterization of AVR-48 NP

Size and Zeta potential of AVR-48 NPs were determined by Brookhaven dynamic light scattering device (DLS, Brookhaven Instruments Co.). Nanoparticle morphology was observed via Hitachi transmission electron microscopy (TEM, H-9500). AVR-48 NPs stability in saline and simulated body fluid (SBF) was confirmed by checking size changes at different time points over 48 hours. Drug loading efficiency and drug release profile were quantified by measuring AVR-48 absorbance (240 nm from 200-1000 spectrum) using Tecan spectrophotometry. For AVR-48 loading efficiency, free AVR-48 was diluted in a similar solvent to the sample solvent at different concentration to make the standard curve. The

supernatant of AVR-48 NPs fabrication was used to measure the amount of unloaded AVR-48. The AVR-48 loading efficiency was calculated by the following formula:

$$AVR - 48\, NP\ loading\ efficiency\ (\%) = \frac{(Initial\ AVR{-}48) - (Unloaded\ AVR{-}48)}{(Inital\ AVR{-}48)} \times 100\%$$

Drug release study from AVR-48 NPs

Standard concentrations of AVR-48 in DI water were prepared and the absorbance was determined at a wavelength of 240 nm from 200-1000 nm spectrum. At 240 nm, a linear fit was obtained to calculate the loading efficiency, drug content and drug release profile of AVR-48 NP. For the drug release study, 4 separate solutions of 5 mg/ml of AVR-48 NP in PBS (pH 7.4) were placed in a dialysis bag with MWCO 3.5-5 kDa (Spectrum, Catalog 131192, Irving, TX, USA), submerged in 20 ml dialysate (1X PBS pH7.4) and incubated at 37°C over a time range (0, 15 min, 30 min, 1h, 2h, 6h, 12h, 1d, 2d, 7 d and 15 d). At each time point, 1 ml of dialysate solution was pooled and replaced with the same volume of fresh PBS. Each sampling solution was then read for its absorbance value and the amount of released AVR-48 was quantified against the AVR-48 standard curve. A cumulative release profile of AVR-48 over time was plotted.

Toxicology Study

The toxicology study was performed on rat pups with 2 SC or IV dose of AVR-48 on postnatal day (P3 for PK Day1) and (P5 for PK Day 3) in two phases following standard FDA guidance [M3(R2)R]. The control/vehicle and test item formulations were injected using insulin syringes (EliMedical, 0.3cc 29G) into the temporal veins of the pups starting on P3, twice a day for a total of 6 doses (P3 to P5) by slow bolus IV injection, according to ITR SOP # VET 46.0. The temporal veins were alternated from one side to the other between each dosing, when feasible. In some instances, the same side of temporal vein was

used due to technical or visual difficulty dosing the other side, or vice versa. Alternatively, the jugular vein was used in a minority of instances. In the tolerability study (ITR Study No. 74595) conducted in juvenile pups (P14), a single administration of AVR-48 at 100 mg/kg/day, IV was well tolerated with no clinical signs of toxicity. Thus, the extravascular SC route of administration, up to 150 mg/kg/day and IV route up to 100 mg/kg/day was considered suitable for this study.

Eighteen animals of each sex were dosed at each dose level. During the repeat dose phase, daily doses of 0, and 100 mg/kg/day for IV and 0, 100, and 150 mg/kg for SC were administered as two divided doses by slow bolus injection at a volume of 5 mL/kg of body weight approximately 6–7h apart. Study animals (n = 5/sex/dose) were treated for three days with a total of six doses for AVR-48. Twelve to eighteen hours after the last dose was administered, animals were euthanized and blood collected for analysis of clinical chemistry (A/G ratio, ALT, Albumin, ALK, AST, bilirubin, calcium, chloride, cholesterol, creatinine, globulin, glucose, phosphorous, sodium, total protein, triglycerides and urea) and other hematological parameters like red blood cell (RBC) count, hematocrit, hemoglobin, white blood cell (WBC) count, WBC differential (absolute), mean corpuscular hemoglobin concentration, morphology of cells, platelet count and reticulocyte count (absolute). Detailed necropsy examinations were performed on each animal, and tissues were collected and fixed with standard fixatives for histological analysis and interpretation

Pharmacokinetic (PK) Study

Two PK studies were conducted – one for mice and the other for rat pups:

In the first study, C57BL/6J mouse pups (P17-P18), pups were dosed IP with AVR-48 solution in PBS (30µl, 10 mg/kg) and AVR-48 nanosuspension (6µl, 0.22mg/kg) in each nostril and euthanized at time points shown in **Table S1** following which blood (plasma) was collected and lungs harvested from each

animal for the above mentioned time points. Two pups were used as controls without any drug administration.

In the second study, rat pups (P3-P5) were dosed either IV (100 mg/kg) or SC (100, 150 mg/kg). Eighteen pups/sex/dose were used for PK analysis and approximately 0.3 ml blood was collected from each pup on days 1 and 3 of the treatment period at time points of 5, 10, 15, 30, 60- minutes post dose. Immediately after dosing (within 4 minutes), the pups were randomized to provide a total of three samples/sex for each time point. Blood was collected by jugular venipuncture into tubes containing the anticoagulant, K_3EDTA. The collection of BAL was limited to the terminal time points of 6h, 12h, and 24h. AVR-48 concentrations in the blood and BAL were determined using LC/MS/MS analysis developed in-house with a dynamic range of 2.0 ng/mL to 1000 ng/mL. PK parameters were estimated using Phoenix pharmacokinetic software (Certara, NJ, USA) using a non-compartmental approach, consistent with the IV bolus injection.

Isolation of pulmonary lymphocytes and flow cytometry

To quantify the immune cells in the lungs dosed with 10 mg/kg AVR-48 (IP), lungs were removed from individual mice at P14 and pulmonary lymphocytes were isolated. The tissue was digested for 2 hours at 37°C with 3.0 mg/ml collagenase A and 0.15 µg/ml DNase I (Roche) in RPMI1640 containing 5% heat-inactivated FBS (Life Technologies, Carlsbad, CA), L-glutamine (2mM), penicillin (100IU/ml), streptomycin (100µg/ml) (Mediatech, Manassas, VA,). The digested tissue was then passed through a 40-µm cell strainer (Falcon) and washed in the same media as above. Cells were counted using trypan blue exclusion with light microscopy.

Flow cytometry was done following the methodology described previously [2]. The single cell suspension was used to identify lymphocytes versus innate immune cells. The lymphocyte panel was co-stained with: anti-mouse CD3 conjugated to APC, CD4 conjugated to Pacific Blue, CD8a conjugated to PE, CD19 conjugated to AF488, and NK 1.1 conjugated to APC Cy7. For the innate immune panel, cells were co-stained with: anti-mouse CD45 conjugated to PerCP Cy 5.5, CD11b conjugated to APC, CD11c conjugated to PE eF610, CD103 conjugated to AF488, MHC-II conjugated to AF700, F4/80 conjugated to PE, Ly6G conjugated to Pacific Blue. The staining was completed with Fc block on ice to prevent internalization and fixed in 1% paraformaldehyde (Fisher Scientific, Hampton, NH) before flow cytometric analysis. The absolute cell numbers were calculated per 100mg of lung tissue. Data were collected on a cell analyzer Fortessa using FACS Diva software (BD Biosciences, San Jose, CA) and analysis was performed using Flow Jo v.10 software (Tree Star).

Supplemental Table-S1: Experimental Design for PK study (C57BL/6J P17-P18 mouse pups)

**Treatment**	**Group 1 (AVR-48) (0.22 mg/kg)**	**Group 2 AVR-48 (10 mg/kg)**	**Group 3 AVR-48 NPs (0.22 mg/kg)**	**Group 4 AVR-48 (0.22 mg/kg)**
**Route/Formulation**	**IP/saline solution**	**IP/saline solution**	**IN/nanosuspension**	**IN/saline solution**
**No. of mouse pups (time point for drawing blood sample)**	3 (5 min)	3 (5 min)	4 (0.5h)*	3 (0.5h)
	3 (15 min)	3 (15 min)	3 (1h)	3 (1h)
	3 (30 min)	3 (30 min)	3 (2h)	3 (2h)
	3 (1h)	3 (1h)	5 (4h)	3 (4h)
	3 (2h)	3 (2h)	3 (8h)	3 (8h)
	-	-	3 (12h)	2 (12h)
	-	-	3 (16h)	3 (16h)
	-	-	3 (24h)	3 (24h)

*4 animals used; 4 lung samples and 3 plasma samples collected (one plasma sample lost in processing)
IP: intra-peritoneal; IN: intra-nasal; min: minutes; h: hours.

Supplemental Table S1A: Pharmacokinetic parameters in neonatal mouse plasma via IP and IN dosing

Matrix	Dose (mg/kg)	Route of dosing/ Formulation	Cmax (ng/mL)	SE Cmax (ng/mL)	Tmax (hr)	$AUC_{(0-t)}$ (hr*ng/mL)	SE $AUC_{(0-t)}$ (hr*ng/mL)	$AUC_{(0-inf)}$ (hr*ng/mL)	$T_{1/2}$ (hr)
Plasma	0.22	IP/solution	14.53*	3.82*	0.083*	3.88*	0.72*	NR	NR
	10	IP/solution	1981.77	312.53	0.083	399.81	46.30	400.46*	0.36*
	0.22	IN/solution	187.21	175.83	1.00	520.06	356.72	NR	NR
	0.22	IN/nanosuspension	NR	NR	NR	NR	NR	NR	NR

* Presented for information purpose, some samples were below the lower level of quantification (LLOQ), but with a signal to noise of 1 to 5.
IP: intra-peritoneal; IN: intra-nasal; hr: hours; SE: standard error; AUC: area under the curve; NR: not reported.

Supplemental Table S1B: Pharmacokinetic parameters in neonatal mouse lungs via IP and IN dosing

Matrix	Dose (mg/kg)	Route of dosing/ Formulation	Cmax (ng/mL)	SE Cmax (ng/mL)	Tmax (hr)	$AUC_{(0-t)}$ (hr*ng/mL)	SE $AUC_{(0-t)}$ (hr*ng/mL)
Lung	0.22	IP/solution	NR	NR	NR	NR	NR
	10	IP/solution	0.25*	0.04*	0.083*	0.04*	0.01*
	0.22	IN/solution	1.12*	0.73*	1.00*	0.28*	0.18*

* Presented for information purpose, samples were below the lower level of quantification (LLOQ), but with a signal to noise of 1 to 5.
IP: intra-peritoneal; IN: intra-nasal; hr: hours; SE: standard error; AUC: area under the curve; NR: not reported.

Supplemental Table-S2: Experimental Design for PK study (rat pups)

Group Numbers	Group Designation	Route of Administration	Dose Level (mg/kg /dose)	Dose Level (mg/kg /day)	Dose Concentration (mg/ml)	Main Animals		Pharmacokinetic Animals
						Male	Female	Male or Female
1	Vehicle*	IV	0	0	0	18	18	0
2	SC-100	SC	50	100	10	18	18	36
3	SC-150	SC	75	150	15	18	18	36
4	IV-100	IV	50	100	10	18	18	36

*The control/vehicle animals received the vehicle (10% DMSO, 20% Tetraglycol and 20% PEG 400 in Purified Water) alone. IV: intravenous; SC: sub-cutaneous.

Supplemental Table S2A: Toxicokinetic parameters in plasma (rat pups) following 50 and/or 75 mg/kg/dose SC or IV bolus administration of AVR-48 on Day 1 (including samples > ULOQ)

Day	Dose (mg/kg)	Route	Tmax (min)	C_0 (µg/mL)	Cmax ± SE (µg/mL)	$AUC_{(0-t)}$ ± SE (min*µg/mL)	$AUC_{(0-t)}$/D (min*µg/mL/ (mg/kg))	$T_{1/2}$ (min)
1	50	SC	30.0	NC	36.4 ± 9.0	1720 ± 372	34.4	NC
	50	IV Bolus	2.00	277.9	196 ± 5.1	5460 ± 384	109.1	NR
	75	SC	60.0	NC	95.0 ± 8.2	2800 ± 294	37.3	NC

ULOQ: Upper level of quantification; IV: intravenous; SC: sub-cutaneous; min: minutes; NC: Not calculated; SE: standard error; AUC: area under the curve.
NR: Result not reported because extrapolation exceeds 20%, or R-squared is less than 0.800.

Supplemental Table S2B: Toxicokinetic parameters in plasma (rat pups) following 50 and/or 75 mg/kg/dose SC or IV bolus administration of AVR-48 on Day 3 (including samples > ULOQ)

Day	Dose (mg/kg)	Route	Tmax (min)	C_0 (µg/mL)	Cmax (µg/mL)	$AUC_{(0-t)}$ (min*µg/mL)	$AUC_{(0-t)}$/D (min*µg/mL/ (mg/kg))	$T_{1/2}$ (min)	R_{AUC} (RATIO)
3	50	SC	30.0	NC	42.2 ± 15.3	1920 ± 371	38.4	NC	1.1
	50	IV	5.00	90.1	154 ± 3.0	4200 ± 572	84	NR	0.8
	75	SC	30.0	NC	85.0 ± 22.1	4060 ± 690	54.1	NC	1.4

ULOQ: Upper level of quantification; IV: intravenous; SC: sub-cutaneous; min: minutes; NC: Not calculated; SE: standard error; AUC: area under the curve.
R_{AUC}: Day 3 $AUC_{(0-t)}$ - Day 1 $AUC_{(0-t)}$.
NR: Result not reported because extrapolation exceeds 20%, or R-squared is less than 0.800.
ULOQ: Upper level of quantification

Supplemental Figures and Legends

Fig. S1

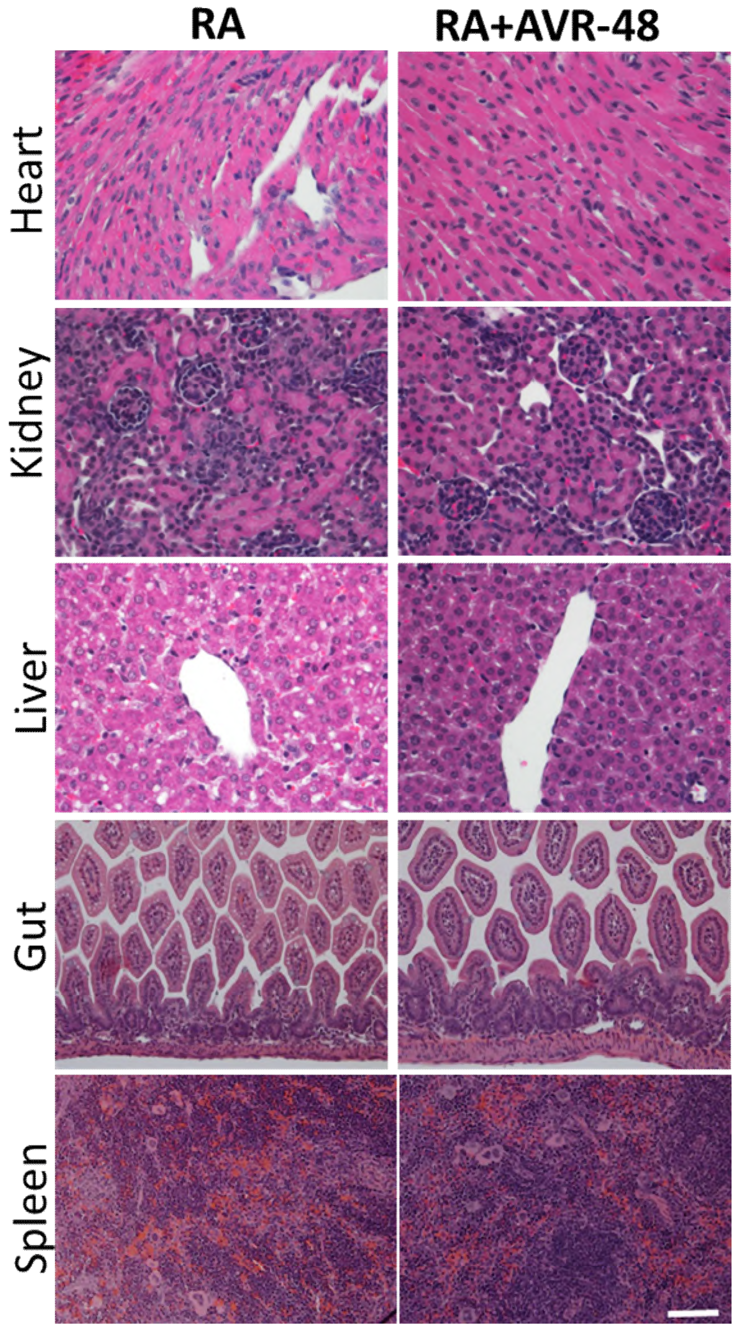

Supplemental Figure S1: **AVR-48 is non-toxic:** Hematoxylin-Eosin staining on histological sections (5µm) of heart, kidney, liver, gut and spleen of P7 mouse pups to show no thrombosis or hemorrhage or edema in these organs when compared with RA controls. RA: room air; scale bar: 100µm. N=3.

Fig. S2

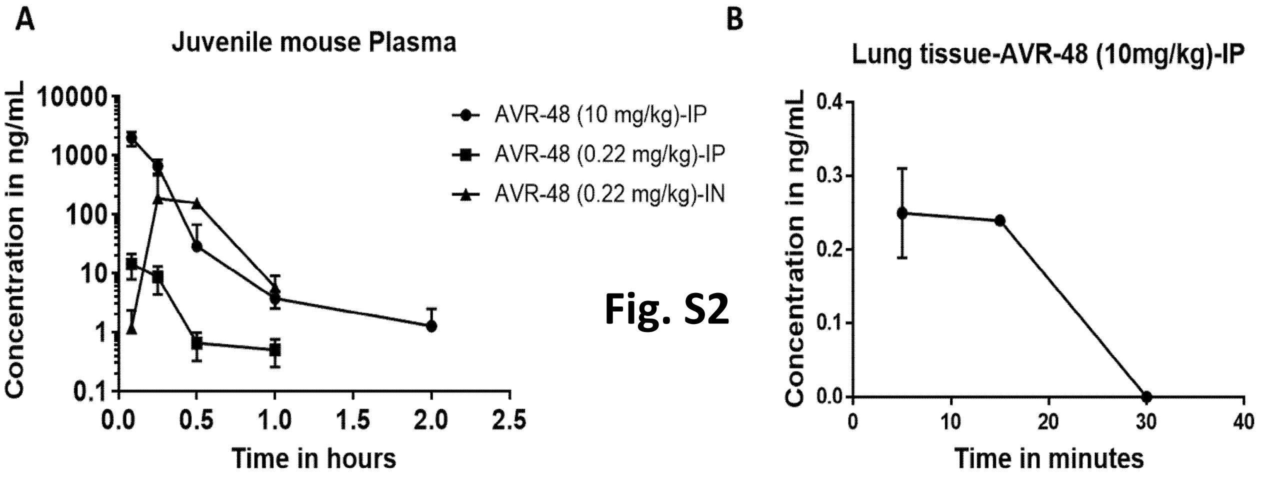

Supplemental Figure S2: **Bioavailability of the drug in mouse pups (A)** Mean AVR-48 concentrations in the plasma. **(B)** Mean AVR-48 concentrations in the lungs after IN or IP administration IP: intraperitoneal; IN: intranasal; N=6.

Fig. S3

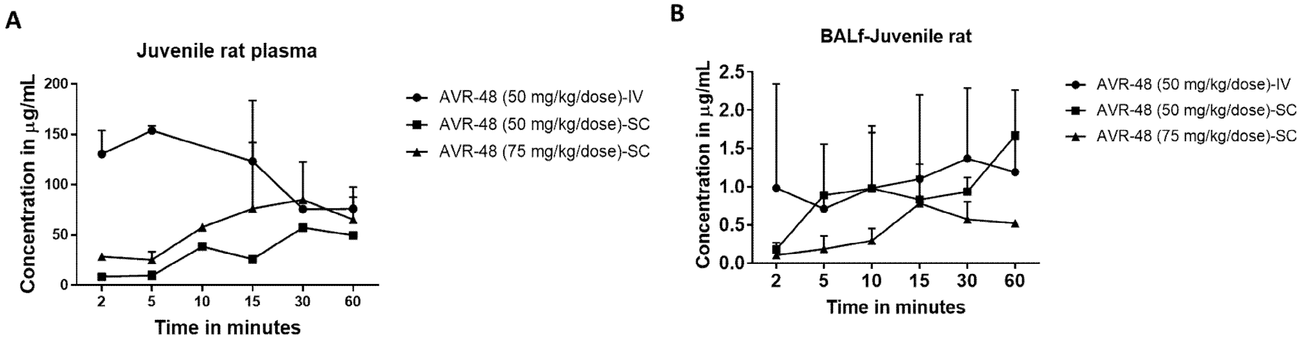

Supplemental Figure S3: **Bioavailability of the drug in rat pups (A)** Mean AVR-48 concentrations in plasma. **(B)** Mean AVR-48 concentrations in the BAL following IV and SC administration. IV: intravenous; SC: subcutaneous; BALf: bronchoalveolar lavage fluid; N=6.

Fig. S4

Compound	Size (nm)	PDI	Zeta potential
AVR-48	369±45	0.28	-19.36

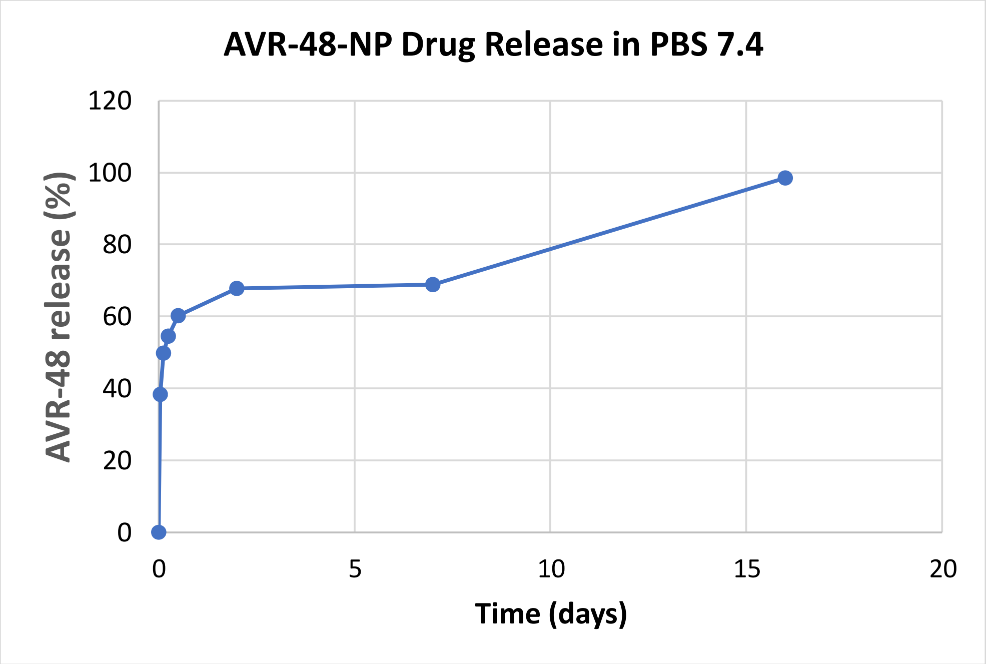

Supplemental Figure S4: **Characterization of AVR-48 nanoparticles and drug release**. The size of the nanoparticle was determined using DLS and was found to be 369±45 nm and zeta potential to -19.36 mV. Drug release study of AVR-48 from its PLGA nano-encapsulated nanosuspension AVR-48 NPs in saline was conducted for 15 days at 37°C and the UV absorbance readings were taken in triplicates at 240 nm. DLS: dynamic light scattering; PLGA: poly D, L-lactic-co-glycolic acid.

Fig. S5

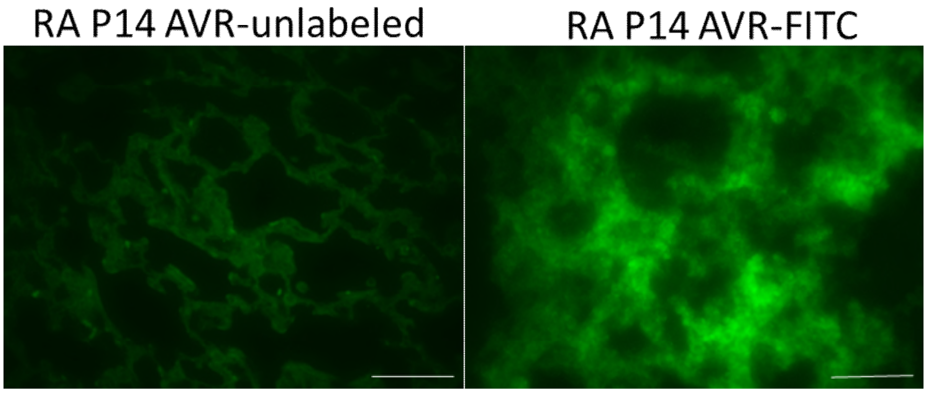

Supplemental Figure S5: AVR compound reaches the lungs when delivered IN. AVR-48 was conjugated with FITC, encapsulated in PLGA similar to the methodology described for AVR-48 NP and delivered IN (20µM). Cryosections of the lungs show **(A)** the absence of the drug with unlabeled compound and **(B)** with the labeled compound. IN: intranasal; RA: room air; FITC: fluorescein isothiocyanate; PLGA: poly D, L-lactic-co-glycolic acid; NP: nanoparticles. Scale bar 100µm, N=2.

Fig. S6

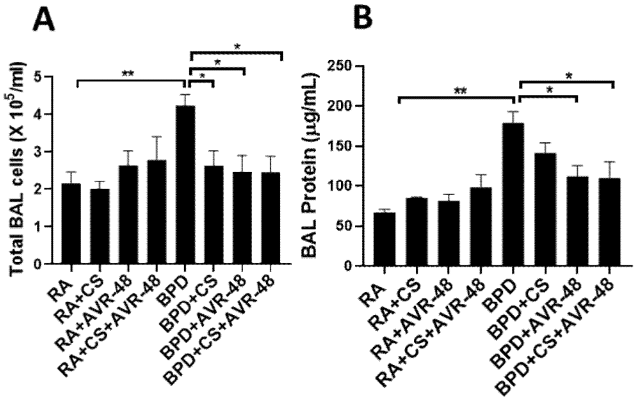

Supplemental Figure S6: AVR-48 is compatible with exogenous surfactant. (A) There was no difference in the total inflammatory cells as well as (**B**) the total protein in the BAL fluid between the BPD group treated with CS alone or with AVR-48 alone or with a combination of CS+AVR-48. *p<0.05; **p<0.01; RA: room air; BPD: Bronchopulmonary dysplasia; CS: Curosurf®, the surfactant used in this study; BAL: bronchoalveolar lavage; N=3-6.

Fig. S7

A

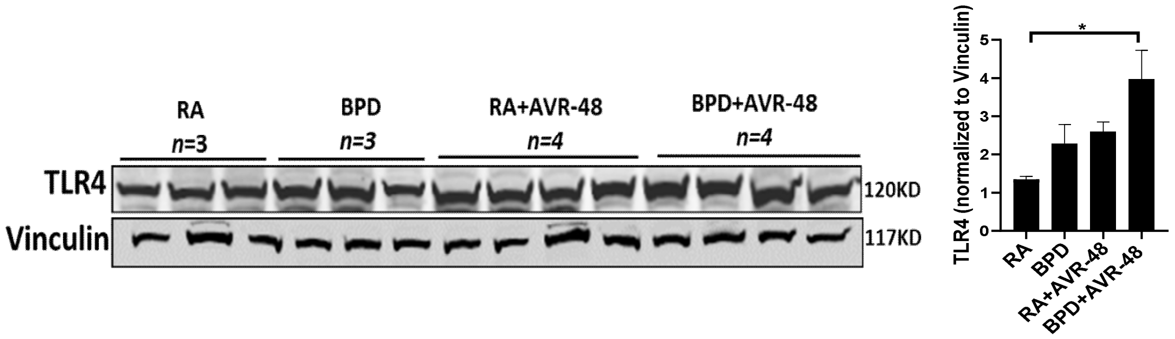
RA
n=3
BPD
n=3
RA+AVR-48
n=4
BPD+AVR-48
n=4
TLR4
Vinculin
120KD
117KD
TLR4 (normalized to Vinculin)
*
RA
BPD
RA+AVR-48
BPD+AVR-48

B

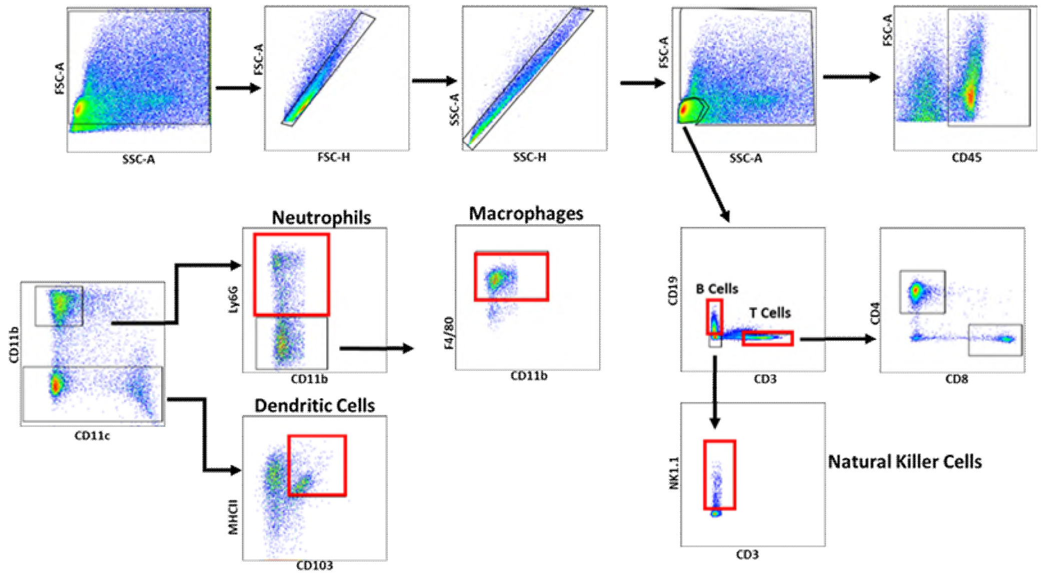
FSC-A
SSC-A
FSC-H
SSC-H
CD45
Neutrophils
Ly6G
CD11b
Macrophages
F4/80
CD11c
Dendritic Cells
MHCII
CD103
CD19
B Cells
T Cells
CD3
CD4
CD8
NK1.1
Natural Killer Cells

C

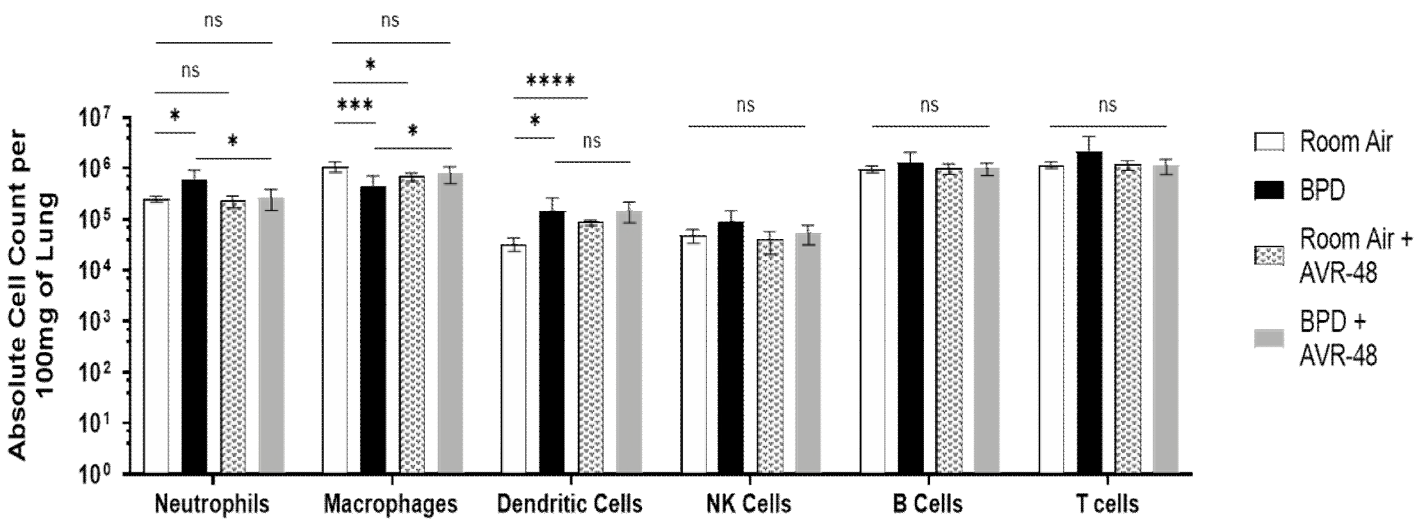
Absolute Cell Count per 100mg of Lung
ns
*
***
****
Neutrophils
Macrophages
Dendritic Cells
NK Cells
B Cells
T cells
Room Air
BPD
Room Air + AVR-48
BPD + AVR-48

Supplemental Figure S7: AVR-48 normalizes two important innate immune cell populations in animals with BPD. (**A**) Representative western blotting of lung homogenates with corresponding densitometric quantification (top right panel) of TLR4. Vinculin is the loading control and is the same one as shown in **Fig. 7A** because the same samples were used. (**B**) Gating strategy used to separate the alveolar immune cell populations by appropriate labelling. (**C**) There was a significant increase in neutrophils and dendritic cells, but a decrease in macrophages, in the BPD versus RA groups in the lung. AVR-48- treated RA animals had a slight, but statistically significant, decrease in macrophages and increase in dendritic cells in the lung, compared to RA animals. AVR-48 treated BPD animals had decreased neutrophils and increased macrophages compared to untreated BPD animals, and these cell populations were at similar levels as the RA control group. $^*p<0.05$; $^{***}p<0.001$; ns: not significant. RA: room air; BPD: Bronchopulmonary dysplasia; TLR: toll-like receptor; N=5-8.

Supplemental Figure S8: All uncut western blot gels shown in the “Results” section.

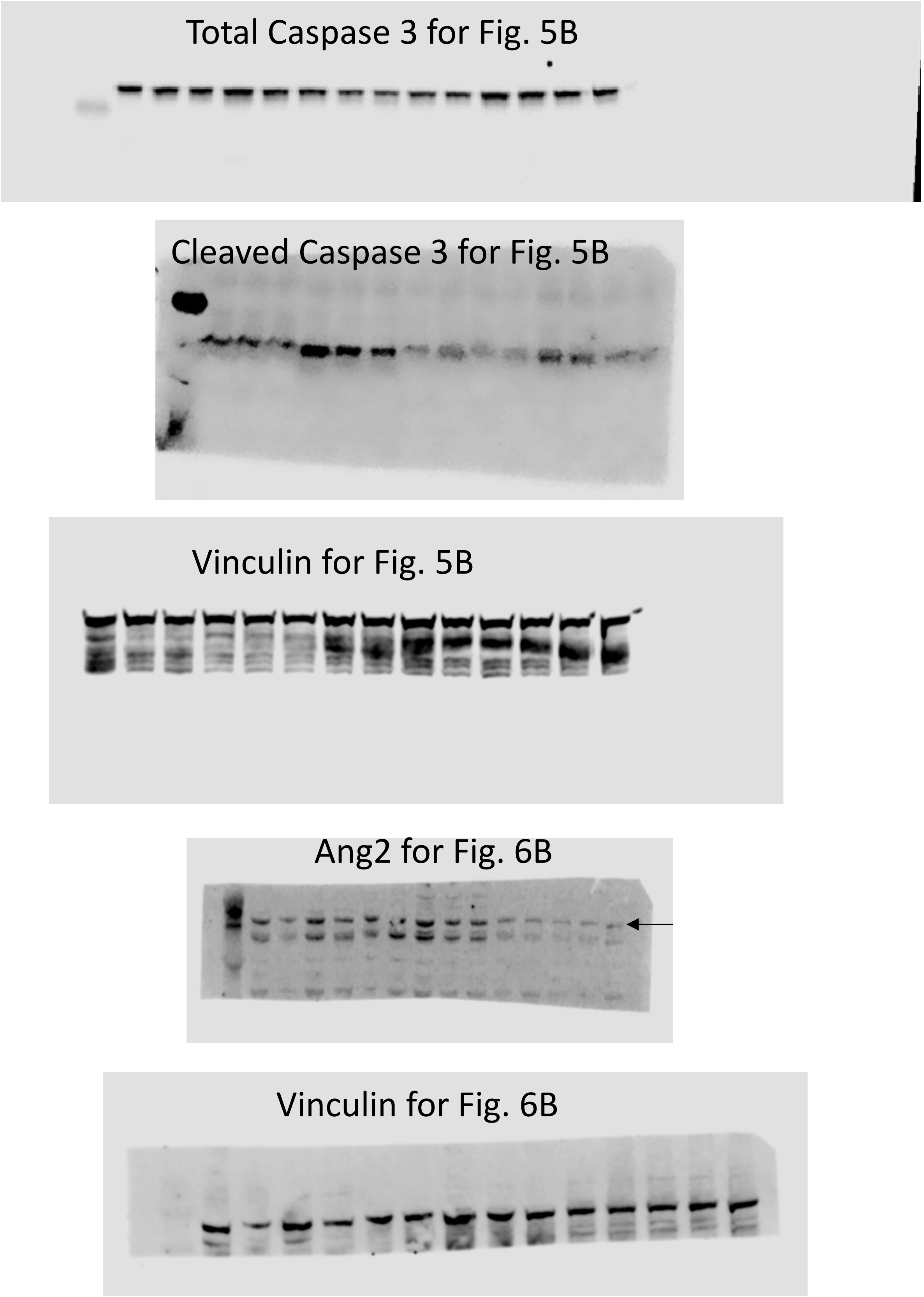

TGFβ for Fig. 7A

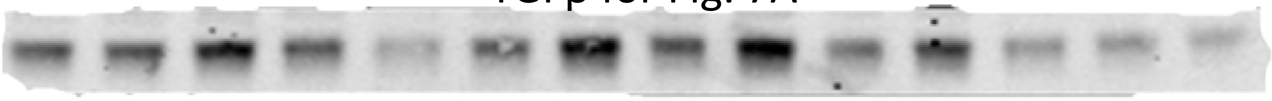

NFkB for Fig. 7A

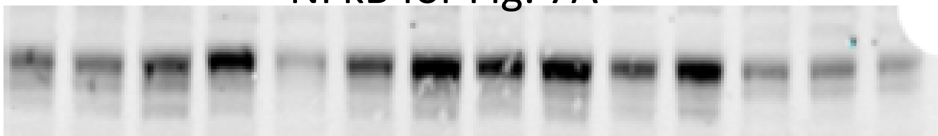

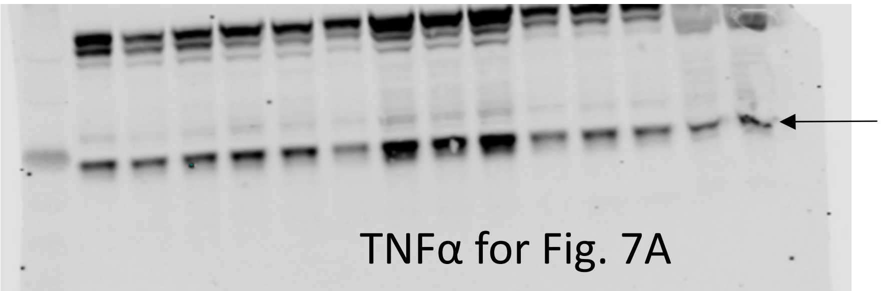

TNFα for Fig. 7A

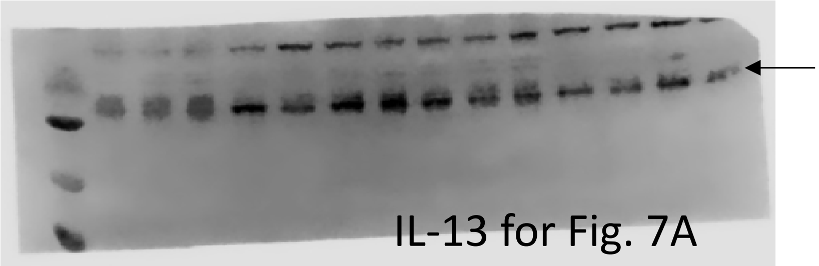

IL-13 for Fig. 7A

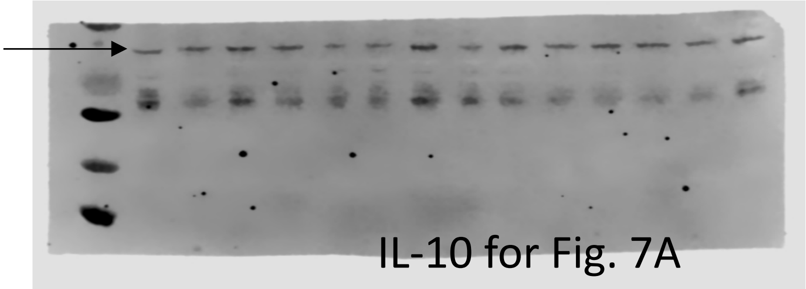

IL-10 for Fig. 7A

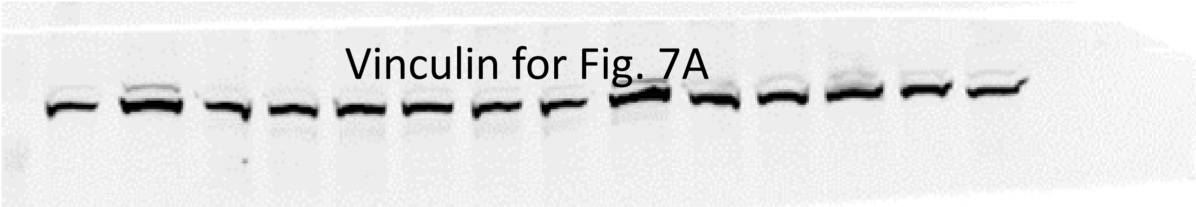

Vinculin for Fig. 7A

IL-1β for Fig. 7B

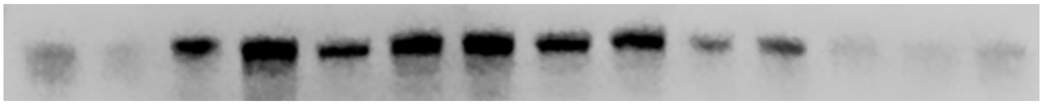

IL-4 for Fig. 7B

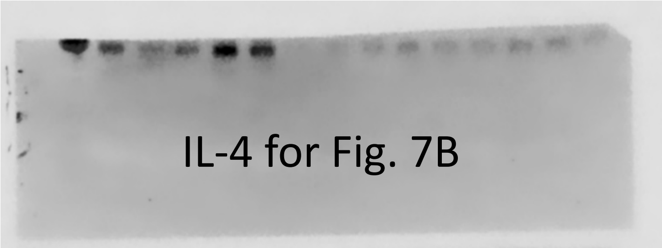

Vinculin for Fig. 7B

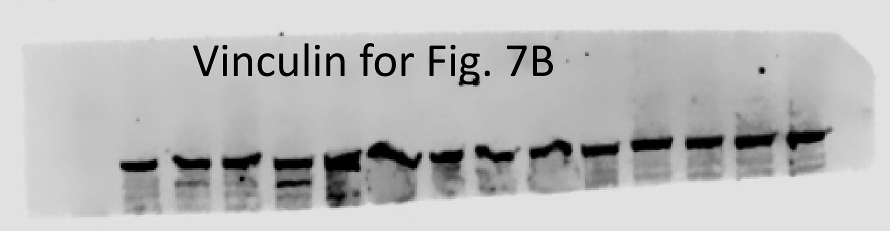

Vegf for Fig. 8B

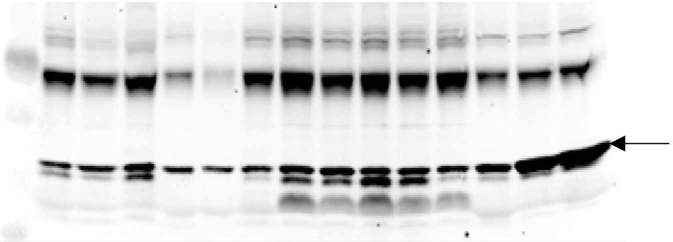

BmpRII for Fig. 8B

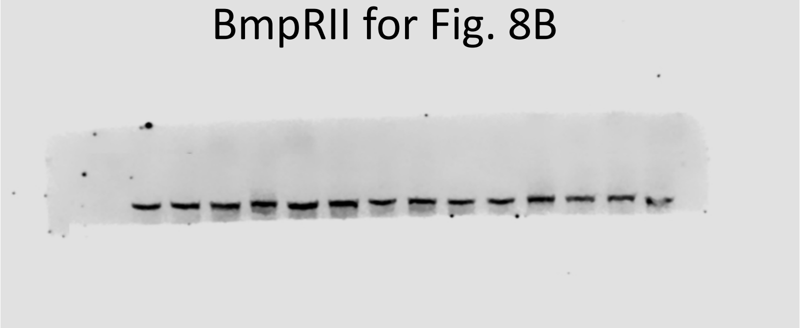

Vinculin for Fig. 8B

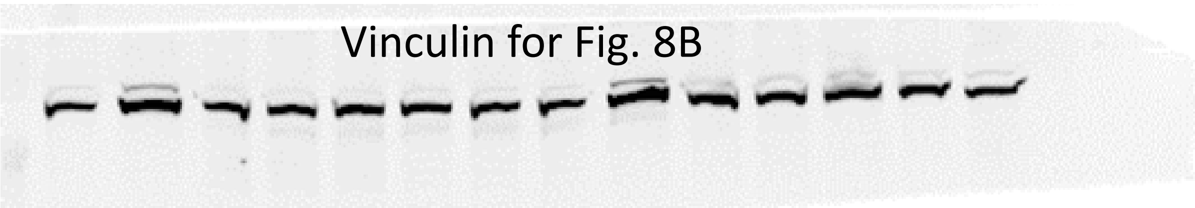

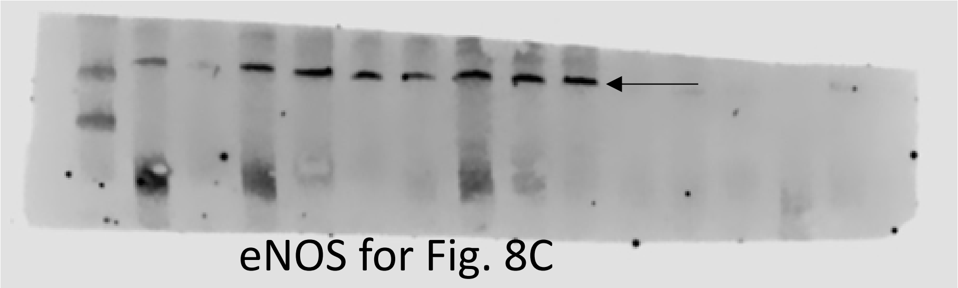
eNOS for Fig. 8C

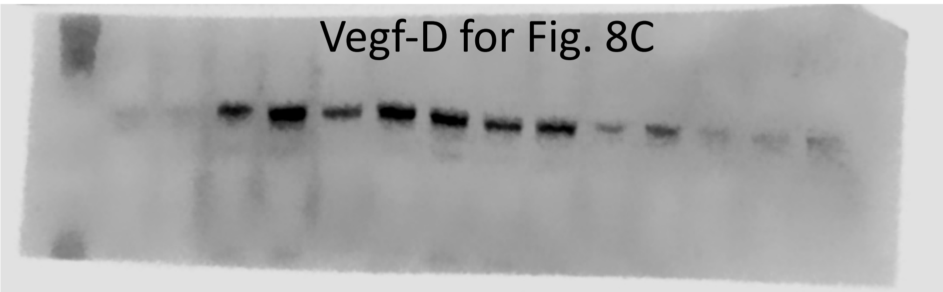
Vegf-D for Fig. 8C

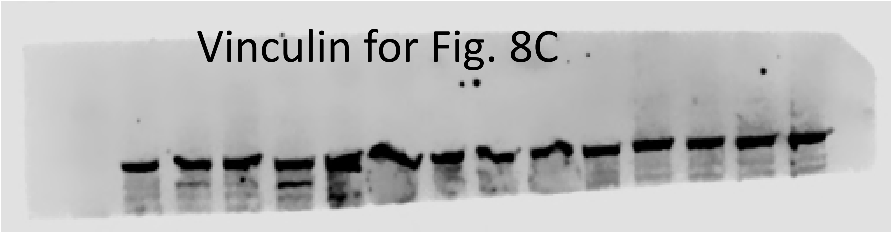
Vinculin for Fig. 8C

TLR4 for Fig. S7A

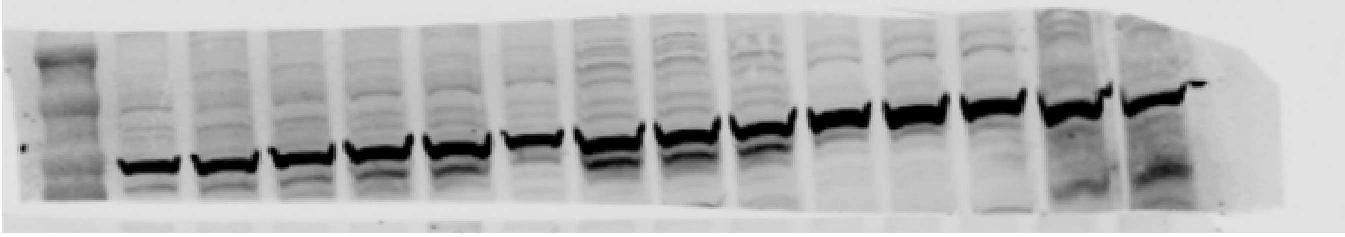

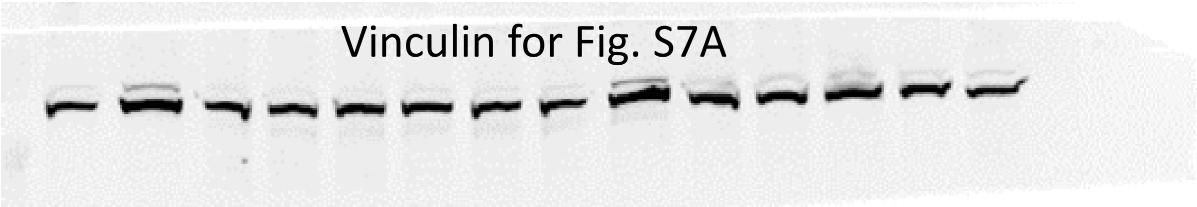
Vinculin for Fig. S7A

References:

1. Le, D.Q., et al., *Hybrid Nitric Oxide Donor and its Carrier for the Treatment of Peripheral Arterial Diseases.* Sci Rep, 2017. **7**(1): p. 8692.
2. Carey, A.J., et al., *Rapid Evolution of the CD8+ TCR Repertoire in Neonatal Mice.* J Immunol, 2016. **196**(6): p. 2602-13.
